# Meshed neuronal mitochondrial networks empowered by AI-powered classifiers and immersive VR reconstruction

**DOI:** 10.3389/fnins.2023.1059965

**Published:** 2023-02-02

**Authors:** Shu-Jiao Li, Hui Liu, Fei-Fei Wu, Da-Yun Feng, Shuai Zhang, Jie Zheng, Lu Wang, Fei Tian, Yan-Ling Yang, Ya-Yun Wang

**Affiliations:** ^1^Specific Lab for Mitochondrial Plasticity Underlying Nervous System Diseases, National Teaching Demonstration Center, School of Basic Medicine, Air Force Medical University (Fourth Military Medical University), Xi’an, China; ^2^Department of Human Anatomy, Histology and Embryology, Medical School of Yan’an University, Yan’an, China; ^3^Department of Neurosurgery, Tangdu Hospital, Air Force Medical University (Fourth Military Medical University), Xi’an, China; ^4^Department of Hepatobiliary Surgery, Xijing Hospital, Air Force Medical University (Fourth Military Medical University), Xi’an, China; ^5^State Key Laboratory of Military Stomatology, School of Stomatology, Air Force Medical University (Fourth Military Medical University), Xi’an, China

**Keywords:** mitochondrial networks, mesh, artificial intelligence, virtual reality, PCs-Mito-GFP mice

## Abstract

Mitochondrial networks are defined as a continuous matrix lumen, but the morphological feature of neuronal mitochondrial networks is not clear due to the lack of suitable analysis techniques. The aim of the present study is to develop a framework to capture and analyze the neuronal mitochondrial networks by using 4-step process composed of 2D and 3D observation, primary and secondary virtual reality (VR) analysis, with the help of artificial intelligence (AI)-powered Aivia segmentation an classifiers. In order to fulfill this purpose, we first generated the PCs-Mito-GFP mice, in which green fluorescence protein (GFP) could be expressed on the outer mitochondrial membrane specifically on the cerebellar Purkinje cells (PCs), thus all mitochondria in the giant neuronal soma, complex dendritic arborization trees and long projection axons of Purkinje cells could be easily detected under a laser scanning confocal microscope. The 4-step process resolved the complicated neuronal mitochondrial networks into discrete neuronal mitochondrial meshes. Second, we measured the two parameters of the neuronal mitochondrial meshes, and the results showed that the surface area (μm^2^) of mitochondrial meshes was the biggest in dendritic trees (45.30 ± 53.21), the smallest in granular-like axons (3.99 ± 1.82), and moderate in soma (27.81 ± 22.22) and silk-like axons (17.50 ± 15.19). These values showed statistically different among different subcellular locations. The volume (μm^3^) of mitochondrial meshes was the biggest in dendritic trees (9.97 ± 12.34), the smallest in granular-like axons (0.43 ± 0.25), and moderate in soma (6.26 ± 6.46) and silk-like axons (3.52 ± 4.29). These values showed significantly different among different subcellular locations. Finally, we found both the surface area and the volume of mitochondrial meshes in dendritic trees and soma within the Purkinje cells in PCs-Mito-GFP mice after receiving the training with the simulating long-term pilot flight concentrating increased significantly. The precise reconstruction of neuronal mitochondrial networks is extremely laborious, the present 4-step workflow powered by artificial intelligence and virtual reality reconstruction could successfully address these challenges.

## Graphical Abstract

Neuronal mitochondrial meshes are the elementary units for neuronal mitochondrial networks (MNs). In the present study, it is the first time to measure two important parameters of the surface area and the volume of neuronal mitochondrial meshes by using artificial intelligence (AI)—powered classifiers and immersive virtual reality (VR) reconstruction. The results show that the surface area (μm^2^) of mitochondrial meshes is the biggest in dendritic trees (45.30 ± 53.21), the smallest in granular-like axons (3.99 ± 1.82), and moderate in soma (27.81 ± 22.22) and silk-like axons (17.50 ± 15.19). And these values show statistically different among different subcellular locations. At the same time, the volume (μm^3^) of mitochondrial meshes is the biggest in dendritic trees (9.97 ± 12.34), the smallest in granular-like axons (0.43 ± 0.25), and moderate in soma (6.26 ± 6.46) and silk-like axons (3.52 ± 4.29). And these values show significantly different among different subcellular locations. 
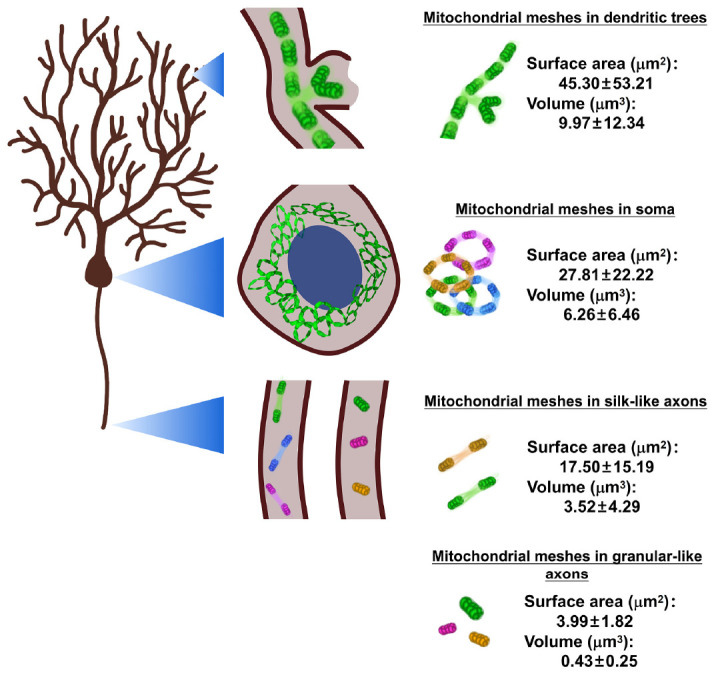

## Highlights

– In this report, we developed a framework to capture and analyze the neuronal mitochondrial networks by using 4-step composed of 2D and 3D observation, primary and secondary virtual reality (VR) analysis, with the help of artificial intelligence (AI)-powered Aivia segmentation an classifiers.
– The 4-step process allowed us to propose that the neuronal mitochondrial networks might be composed by regular mitochondrial meshes, and more importantly, provide the detailed quantitative measurement about the two important parameters of the surface area and the volume about neuronal mitochondrial meshes, by using the generated PCs-Mito-GFP mice in which the mitochondria specific within the cerebellar Purkinje cells are labeled by green fluorescence protein (GFP).
– The results showed that both the surface area and the volume of mitochondrial meshes in Purkinje cells were the biggest in dendritic trees, the smallest in granular-like axons, and moderate in soma and silk-like axons.
– Moreover, both the surface area and the volume of mitochondrial meshes in dendritic trees and soma within the Purkinje cells in PCs-Mito-GFP mice, who were trained by the simulating long-term pilot flight concentrating, showed significantly increased.
– Hence we propose that the AI-powered segmentation and classifiers, combined with the immersive VR reconstruction could resolve the complicated neuronal mitochondrial networks into discrete and quantifiable mitochondrial meshes, so as to achieve the detailed morphological analysis of neuronal mitochondrial networks.

## 1. Introduction

Mitochondria were discovered in the late nineteenth century and were described as a collection of granules forming threads inside the cell (Benda, 1898). In specimens of non-neuronal cells such as endothelium, mesenchyme, giant cells, ectoderm, heart muscle, smooth muscle, and endoderm, mitochondria were present in multiple forms of granules, rods, threads, loops, and networks, and further arranged in a continuous series (Lewis and Lewis, 1914). This view was confirmed by electron-microscopic observations of tissue, cultured cells, and rat diaphragm muscle that revealed the existence of a mitochondrial networks (Lewis and Lewis, 1914). A mitochondrial network is defined as a continuous matrix lumen whose boundaries limit molecular diffusion (Twig et al., 2006; Burté et al., 2015; Moore et al., 2016). Mitochondria do not exist as isolated organelles; instead, they form a highly interconnected tubular network throughout the cell (Burté et al., 2015). However, investigation into the morphological properties of mitochondrial networks has not yielded consistent conclusions.

Observation of individual networks has proven challenging in neuronal cells that possess dense populations of mitochondria. Neurons (Harris et al., 2012) are morphologically complex, long-lived, and energetically expensive. Thus, neurons have to maintain mitochondrial networks that are at once stable and tunable, capable of supplying energy to distant synapses for tens of years while also adapting to fluctuating energy demands. A properly organized, healthy mitochondrial network is critical for preserving neuronal form and function (Barnhart, 2016). Large, elaborately branched neuronal morphologies, energetic demands that fluctuate in time and space, and long neuronal lifespans make the distribution of mitochondria in neurons a particularly complex problem (Nunnari and Suomalainen, 2012; Youle and van der Bliek, 2012; Anzell et al., 2018). Studies typically utilize a qualitative or semi-quantitative approach by developing a scoring system of fission/fusion profiles or qualitatively evaluating mitochondria into categories of “fused,” “fragmented,” and “intermediate” morphologies, both of which lack precise assessment of mitochondrial network morphologies (Kumar et al., 2016; Prieto et al., 2016; Tang et al., 2016). Machine learning is a statistical and computational technique that may be used to derive a classification scheme from classified training data (Leonard et al., 2015). Every individual mitochondrion can be classified with the ctree method from a published computational toolbox for conditional inference recursive partitioning^1^ in R version 3.0.1 (64-bit) (Leonard et al., 2015). The Mitochondrial Network Analysis (MiNA) toolset is a relatively simple pair of macros making use of existing ImageJ plug-ins, allowing for semi-automated analysis of mitochondrial networks on two-dimensional (2D) images (Song et al., 2008). Three-dimensional (3D) imaging and quantification are crucial for proper understanding of mitochondrial shape and topology in think neuronal cells (Mitra and Lippincott-Schwartz, 2010; Chevrollier et al., 2012; Lihavainen et al., 2012; Nikolaisen et al., 2014; Tronstad et al., 2014). Integrative 3D analysis of mitochondrial network properties has been found to provide new insight into important aspects of mitochondrial dynamics and neuronal function. This paper discusses a technology that makes light microscope data oriented manual reconstruction more efficient and reliable than existing approaches. This work was motivated by three purposes detailed below: (1) hierarchical streaming of teravoxel-scale images of adjacent and intertwined mitochondrial structures, (2) immersive and collaborative 3D visualization, and (3) interaction.

Whole-cerebellar Purkinje cells reconstruction of mitochondrial network is such challenging as it involves processing tens of teravoxels of imaging data. The large and uniform Purkinje cells are named after the Czech physiologist Purkinje who described them in a paper on the histology of the nervous system presented in Prague in 1837 (Herndon, 1963). Purkinje cells are the sole output neuron in the cerebellar circuit and they arborize highly intricate dendrites that receive thousands of synaptic inputs on their fanshaped dendrites from parallel fibers and climbing fibers (Napper and Harvey, 1988). Purkinje cells thus strategically distribute abundant mitochondria throughout their dendrites, somatic bodies and axons (Fukumitsu et al., 2016). The mitochondria in somatic bodies of Purkinje cells are commonly between 0.75 and 3 μm^2^ in area, similar to those seen in other neurons (Dempsey, 1956; Hartmann, 1956). They are generally sausage-shaped and in most instances the cristae run at right angles to the long axis. Occasional irregular and branched forms are seen. Tubular mitochondria also occupy the primary dendrites, whereas focal clusters of smaller mitochondria appear in the secondary and tertiary dendrites (Pham et al., 2012). The mitochondria in the smaller dendritic branches, on the other hand, tend to be more elongated and their cristae usually run parallel to the long axis of the mitochondrion. These elongated mitochondria are often helpful in identifying the smaller dendritic branches of the Purkinje cells. It is hard to track thin Purkinje cells axons, which makes it more difficult to capture the internal mitochondrial tracks.

The aim of the present study is to develop a framework to capture and analyze the neuronal mitochondrial networks by using 4-step process composed of 2D observation, 3D observation, and followed by primary and secondary artificial intelligence (AI)-powered virtual reality (VR) reconstruction. In order to fulfill this purpose, we first generated the PCs-Mito-GFP mice, in which GFP could be expressed on the outer mitochondrial membrane specifically on the cerebellar Purkinje cells (PCs), thus all mitochondria in the giant neuronal soma, complex dendritic arborization trees and long projection axons of Purkinje cells could be easily detected under a laser scanning confocal microscope. The 4-step process allowed us to resolve the complicated neuronal mitochondrial networks into discrete neuronal mitochondrial meshes. More importantly, we measured the two important parameters of the neuronal mitochondrial meshes. The results showed that the surface area of mitochondrial meshes was the biggest in dendritic trees, the smallest in granular-like axons, and moderate in soma and silk-like axons. And these values show statistically different among different subcellular locations. At the same time, the volume of mitochondrial meshes is the biggest in dendritic trees, the smallest in granular-like axons, and moderate in soma and silk-like axons. And these values show significantly different among different subcellular locations. Finally, we compared both the surface area and the volume of mitochondrial meshes in dendritic trees and soma within the Purkinje cells in PCs-Mito-GFP mice with or without receiving the training with the simulating long-term pilot flight concentrating model. The results showed that the surface area and volume of mitochondrial mesh in dendritic trees or soma of Purkinje cells in PCs-Mito-GFP mice with the training increased significantly, compared with that in mice without the training, by approximately 27 and 38%, respectively. The precise reconstruction of neuronal mitochondrial networks is extremely laborious, the present 4-step workflow powered by artificial intelligence and virtual reality reconstruction could successfully address these challenges.

## 2. Materials and methods

### 2.1. Construction of the PCs-Mito-GFP mouse line

**FIGURE 1:**
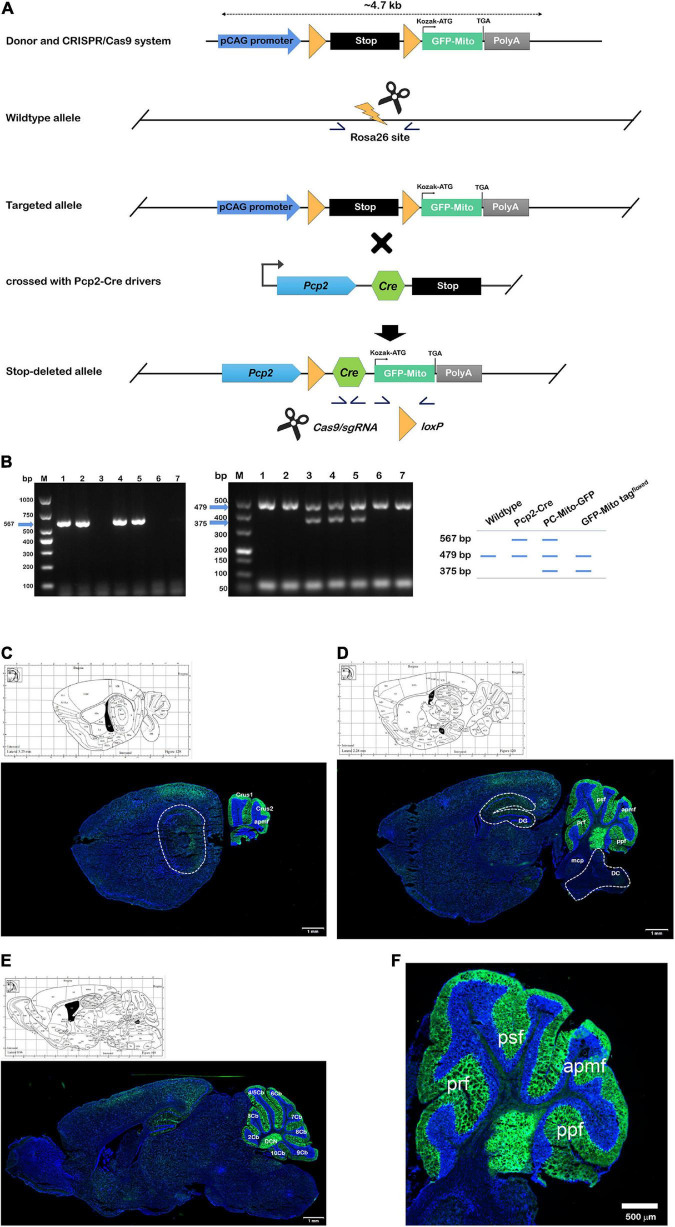
Construction of GFP-Mito tag^floxed^ and PCs-Mito-GFP mouse lines. **(A)** Design and generation of PCs-Mito-GFP mice by using CRISPR/Cas9 system. Homologous recombination of the targeting construct in embryonic stem cells results in insertion of the Cre-dependent mito-GFP cassette into the Rosa26 locus. Then targeting of mito-GFP into the Rosa26 locus. In targeted allele mice, removal of the neomycin selection marker by Flp recombinase results in the PCs-Mito-GFP line, which can be mated to a Cre driver line to obtain cell-specific labeling of mitochondria. Germline excision of the termination signal produces the PCs-Mito-GFP line. Yellow arrowheads, loxP sites; stop symbol, termination cassette; gray diamonds, frt sites; half arrows, PCR primers for genotyping; short horizontal line, probe for Southern blot. **(B)** PCR genotyping of the wildtype, Pcp2-Cre, Pcp2-Mito-GFP and GFP-Mito tagfloxed strain using the three primers in schematic. **(C–E)** The internal image at lateral 3.25 mm **(C)**, 2.28 mm **(D)**, and 0.96 mm **(E)** according to Allen map (http://mouse.brain-map.org/static/atlas). All mitochondria within the PCs in Crus 1, Crus 2, apmf, prf, psf, ppf, as well as 2∼10 Cb of cerebellar cortex expressed bright green fluorescence under a Zeiss confocal microscope equipped with Airyscan 2 system, while all other mitochondria all over the brain and cerebellum had no fluorescence. 2∼10 Cb; the 2nd∼10th cerebellar lobule; apmf, ansoparamedian fissure; Cop, copula of the pyramis; CPu, caudate putamen (striatum); Crus 1/2, crus 1/2 of the ansiform lobule; DC, dorsal cochlear nucleus; DCN, deep cerebellar nucleus; DG, dentate gyrus; mcp, middle cerebellar peduncle; PM, paramedian lobule; ppf, prepyramidal fissure; prf, primary fissure; psf, posterior superior fissure; Sim, simple lobule. Bars in panels **(C–E)** = 1 mm. **(F)** Enlarged version of image d within the cerebellum. Bar = 500 μm.

We first generated the PCs-Mito-GFP mice, in which GFP could be expressed on the outer mitochondrial membrane specifically on the cerebellar Purkinje cells (PCs). As shown in Figure 1A, Pcp2-ires-Cre mice express Cre recombinase under the control of the mouse Purkinje cell protein (Pcp2) and Cre recombinase expression is detected in Purkinje cells (PCs) of the cerebellar folia. MitoTag (Rosa26-CAG-LSL-GFP-OMM) is a Gt(ROSA)26S or knock-in allele that has a loxP-flanked STOP cassette preventing transcription of an outer mitochondrial membrane-targeted enhanced green fluorescent protein (GFP). When crossing the two mice lines, we could obtain the mice with robust GFP fluorescence localizing to mitochondria of PCs faithfully. Pcp2-Cre mice were hybridized with Mito-GFP mice to generate PCs-Mito-GFP mice (Zhang et al., 2004). The offspring mice with both Cre recombinase and Mito-GFP sequences were identified by PCR.

### 2.2. Confirmation of the PCs-Mito-GFP mouse line

To genotype the PCs-Mito-GFP allele and PCs-Mito-GFP by PCR, the set of primers of Cre were used with F3: 5′-ATTCTCGTGGAACTGGATGG-3′,5′-GGACAGGTAATGGTTGTCTGG-3′, resulted with 567 bp product, under the procedures of 94°C 3 min, 94°C 30 s/62°C 35 s/72°C 35 s with 35 cycles, and 72°C 5 min. To genotype the wild-type allele and GFP-Mito tag^floxed^ allele, the set of primers were used with wildtype F1: 5′-CCCAAAGTCGCTCTGAGTTGTTA-3′, wildtype R1: 5′-TGGCGTTACTATGGGAACATACGTC-3′ and insert F2: 5′-CCCAAAGTCGCTCTGAGTTGTTA-3′, insert F2: 5′-TCGGGTGAGCATGTCTTTAATCT-3′, resulted with two products of 479 bp and 375 bp, under the procedures of 95°C 5 min, 98°C 30 s/65°C 30 s (–0.5°C/cycle)/72°C 45 s/98°C 30 s/55°C 30 s/72°C 45 s with 20 cycles, and 72°C 5 min. As shown in Figure 1B, so the wild-type allele yields a 479 bp band, the Pcp2-cre allele yields two bands of both 567 and 479 bp, the GFP-Mito tag^floxed^ allele yields two bands of both 479 and 375 bp, and the target PCs-Mito-GFP allele yields three bands of 567, 479, and 375 bp. As shown in Figures 1C–F, in PCs-Mito-GFP mice, only PCs in the cerebellar cortex exhibited bright mito-GFP fluorescence localized specifically to the mitochondrial compartment.

### 2.3. The framework of 4-step process

**FIGURE 2:**
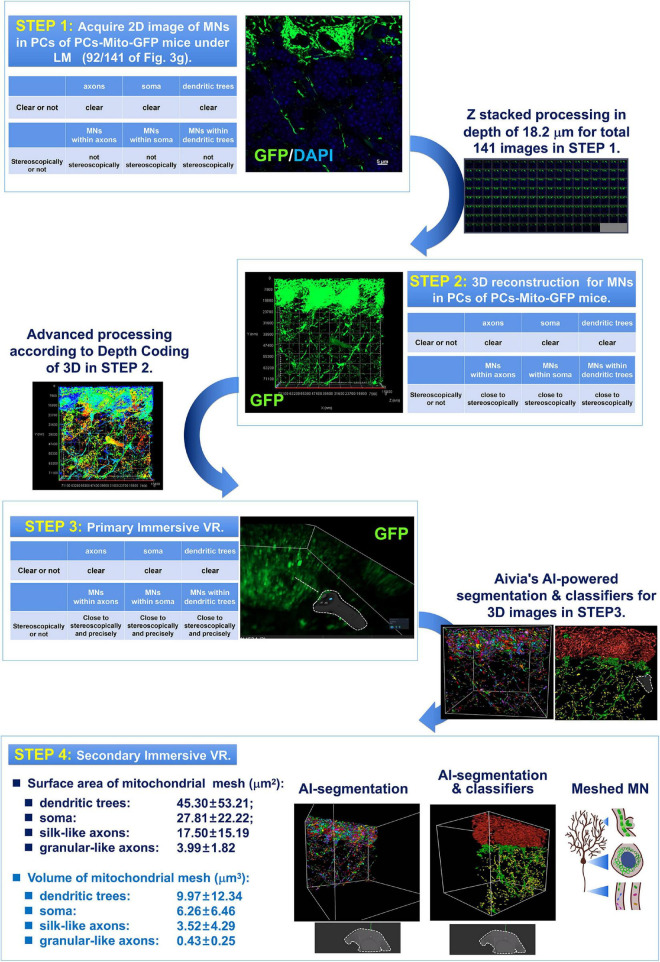
Four step process composed of 2D and 3D observation as well as primary and secondary AI-powered VR were used for detection of the MNs in PCs under light-microscopy (LM). The 1st step was to acquire 2D image of MNs in PCs of PCs-Mito-GFP mice under LM. In the 1st step, although the images of axons, or soma, or dendritic trees were all clear, the MNs within axons, or soma, or dendritic trees could not be indicated stereoscopically and precisely. Then after Z stacked processing in depth of approximately 20 μm for total more than 80 2D images in STEP 1, 3D reconstruction for MNs in PCs of PCs-Mito-GFP mice were made. In the 2nd step, the MNs within axons, or soma, or dendritic trees could be indicated close to stereoscopically. Then after advanced processing according to Depth Coding of 3D, in the Step 3 of primary immersive VR, MNs within axons, or soma, or dendritic trees could be indicated close to stereoscopically and precisely. Finally after Aivia’s AI-powered segmentation and classifiers for 3D images obtained in Step 3, we have stepped into the 4th step, in which we have for the first time found that neuronal MNs were composed by regular meshes. The surface area (μm^2^) of mitochondrial meshes was the biggest in dendritic trees (45.30 ± 53.21), the smallest in axons (17.50 ± 15.19), and in the middle in soma (27.81 ± 22.22). In addition, the volume (μm^3^) of mitochondrial meshes was the biggest in dendrits (9.97 ± 12.34), the smallest in axons (3.52 ± 4.29), and in the middle in soma (6.26 ± 6.46).

As shown in Figure 2, there were four steps in the present study.

#### 2.3.1. Step 1: 2D images of MNs

The first step was to acquire two-dimensional (2D) images. Firstly, we acquired the two-dimensional images, which were prepared under confocal microscopy (LSM900, Zeiss, Germany). Aged 7 weeks PCs-Mito-GFP mice were perfused transcardially with phosphate buffered saline (PBS) followed by 4% fresh formulated paraformaldehyde (Sigma). Tissues were embedded overnight at 4°C in 30% sucrose solution. The cerebellum were isolated and frozen in OCT for sagittal sectioning by a cryostat (CM 1950, Leica, Germany) with 15 μm thickness, which as incubated with DAPI (28718-90-3; Solarbio Co., Ltd., China) for 5 min after washing in PBS. The raw z-stack images of PCs-Mito-GFP were acquired on a Zeiss LSM900 laser scanning confocal microscope equipped with Airyscan 2 system, and a 63 × 1.4 NA Oil DIC M27 objective (Zeiss). We have identified anatomical structures according to Allen map.^2^ For super-resolution microscopic images of Purkinje cells, Z-stack of axons, soma and dendritic trees were 141 slices (18.2 μm) at 0.13 μm interval, 93 slices (13.8 μm) at 0.15 μm interval and 89 slices (11.44 μm) at 0.13 μm interval, respectively.

#### 2.3.2. Step 2: 3D images of MNs

The step 2 was to construct 3D structure based on the multiple 2D images. Three dimensional (3D) images of mitochondrial networks of cerebellar Purkinje cells were composed of all continuous 2D images of Z-stack by Aivia (version 10.5.1, LeiCa, Germany) (Harada et al., 2012; Hintiryan et al., 2021). Aivia uses artificial intelligence technology to register images according to fluorescence intensity and sample size. And each 2D image of Z-stack was reconstructed in spatial order to simulate the actual spatial location of mitochondria in Purkinje cells. Through the 3D view, we can accurately observe the spatial distribution of mitochondria in Purkinje cells, avoiding the visual difference caused by different focal in 2D view.

#### 2.3.3. Step 3: Primary immersive VR observation

We used virtual reality (VR) technology for immersive observation of a continuous matrix lumen shaped mitochondria networks in Purkinje cells. The VR environment was installed the, VIVE from https://www.vive.com/cn/setup/pc-vr/, Steam from https://store.steampowered.com/about/ and SteamVR. The VR was achieved on the support of Aivia software (version 10.5.1, LeiCa, Germany) from https://www.leica-microsystems.com.cn/cn/products/microscope-software/p/aivia/. ziviaVR was implemented and evaluated on computers with Intel(R) Core(TM) i9-10980XE CPU@3.00GHz, 256 GB RAM, 8TB ROM, NVIDIA Quadro RTX 6,000 GPU, Windows 10 64-bit edition, and Aivia Pro as the VR device. And this AiviaVR helped the controller to control the observation view at will and observe mitochondrial networks in an immersive and all-round way.

Before the 4th step, the segmentation and classifiers were performed by using Aivia, and then the continuous matrix lumen shaped mitochondria networks could be converted to discrete neuronal mitochondrial meshes. It should be indicated that Aivia uses an artificial intelligence (AI)-based software architecture to build a complete platform for two-dimensional to five-dimensional image visualization, analysis and data interpretation that reliably processes and reconstructs highly complex images in just a few minutes. AI-powered segmentation is based on different convolutional neural network architectures (DenseNet, UNet, 3D-Unet) to process images. And AI-powered classification, employing object classifier, is in terms of random forest. The Aivia’s AI is characterized by enabling complex, difficult and time-consuming image processing to be completed quickly, objectively and repeatably and efficiently, even when the analyst does not have the relevant expertise. AI is able to reliably process and reconstruct highly complex images in just a few minutes. Thus, reliable and repeatable segmentation results are generated to effectively and quickly realize 2D and 3D image visualization and analysis.

To achieve image visualization rapidly and analyze it accurately and repeatably, we used AI-powered Aivia software to view and analyze our mitochondrial networks. In the present procedures, to quantify the mitochondria, AI-powered segmentation, the module of Recipe Analysis, was used to mesh it, of which the steps followed Aivia’s specification of the recipes analysis module. The whole segmentation process includes two parts: detection and partition. In order to make the data reproducible, we unified the parameters of detection and partition. When detecting mitochondria, the average object radius and minimum edge fluorescence intensity were controlled within 0.7 microns and 5. And in the mitochondrial partition stage, the object radius ranged from 0.2 microns to 50 microns. The minimum distance from the center of an object to the edge that is touching its closet neighboring object was set as 20 microns. With the accurate pinpointing capability in AiviaVR (Supplementary Figures 2–4), in real time a user can precisely and efficiently load the data of a desired high-resolution ROI to see detailed 3D morphological structures (Supplementary Figures 2–4). The data handling of AiviaVR has been engineered to be scalable so that the large amount of volumetric data is no longer a barrier. After AI-powered segmentation, the continuous mitochondria networks within the purkinje cells were converted to discrete neuronal mitochondrial meshes by AI-segmentation.

Next, Aivia AI learning was used to classify the discrete neuronal mitochondrial meshes according to the locations of soma, dendritic trees, axons. And axons were further divided into granular-like axons and silk-like axons. The meshes keeping the same position coordinates and morphological characteristics, including superficial area, volume and immunofluorescence intensity, were marked as soma “Class” through the function of AI teaching of Aivia. The module of “AI-classifier,” an object classifier, was run, of which the operation procedures were conformed to the instruction of Aivia. In our study, meshes were regarded as the object of classification. For example, we selected some meshes from the soma of Purkinje cells and incorporated them into the soma “Class,” which was a process of training. So did the meshes from dendrites and axons of Purkinje cells. Finally, the different colors are used to label meshes in different parts of Purkinje cells.

#### 2.3.4. Step 4: Secondary immersive VR observation

Next the meshed mitochondrial networks achieved by Aivia’s AI-powered segmentation and classifiers were observed by the secondary immersive VR, specially paying attention to the locations of meshes. The secondary VR was achieved successfully by AiviaVR, and the procedures were strictly performed and complied with the instruction of Aivia. The highly complex meshes could be effectively and immersively observed using AiviaVR. We can use the VR controller to control the observation view at will, so as to observe meshes in an immersive and all-round way.

### 2.4. Centrifuge simulating long-term pilot flight

To simulate the condition of a pilot with 1-year-flight, the small animal overweight simulated centrifuge machine (designed by the Fourth Military Medical University; produced by Hunan Kecheng Instrument and Equipment Co., Ltd., Hunan, China, FXCZ-Y1) was used in our experiment, as shown in Supplementary Figure 1. Supplementary Figures 1A–E have shown the main technical parameters: a, containing four mice cabins with 20 cm of centrifugation radius; b, 1∼10 s duration for centrifugal gravity acceleration from 0 to 2∼20 g; c, the centrifuge can be kept stable to 1 g before and after starting the speed up; d, the vacuum pressure value ranged from 0 to 100 Kpa to simulate the plateau environment; e, oxygen concentration in the cavity can be detected and adjusted. Supplementary Figure 1F has shown the centrifuge protocol provided by Professor Da-Yun Feng and Professor Si-Wei Wang, who are all from the Fourth Military Medical University, Xi’an, China. On the first day, the mice were placed in the overweight centrifuge machine. In the first step, the centrifugal force increased from 0 to 1 g in 3 s and lasted for 10 min at 1 g. In the second step, the centrifugal force increased from 1 to 12 g in 4 s and lasted for 10 s at 12 g. In the third step, the centrifugal force decreased from 12 to 1 g in 5 s and lasted for 10 min at 1 g. Step 2 and Step 3 were repeated continuously five times. And, on the second day, the experiment was performed according to the procedures of the first day. In the following 5 days, we performed the experiment in 3 steps every day and the first step was similar to the first 2 days. However, in the second step, the centrifugal force increased from 1 to 14 g in 4 s and lasted for 10 s at 14 g. In the third step, the centrifugal force decreased from 14 to 1 g in 5 s and lasted for 10 min at 1 g. Likewise, steps 2 and 3 were repeated continuously five times without interval. The whole experiment lasted 1 week.

### 2.5. Statistical analysis

The data were expressed as mean ± standard deviation. First, all data were tested for normality and homogeneity of variance test. One-way ANOVA was used to evaluate the differences between the three groups for comparison, and LSD *post-hoc* test was used for multiple comparison between groups. The two samples were compared using the two-tailed Student’s *t*-test. The Kruskal-Wallis test or Mann-Whitney *U*-test were used to analyze the data that did not conform to the test of normality and homogeneity of variance. Graphpad was used for statistical analysis. *P* < 0.05 was considered statistically significant. All scatter graphs and line graphs were created and synthesized by Graphpad software.

## 3. Results

### 3.1. Construction of GFP-Mito tag^floxed^ and PCs-Mito-GFP mouse lines

As shown in Figure 1A, we have designed and generated Rosa26-CAG-LSL-GFP-Mito tag (GFP-Mito tag^floxed^) mouse line (Number: PO-GJS2020062243-01; GemPharmatech Co., Ltd., Nanjing, China), in which a mitochondrial localized version of the enhanced green-fluorescence-protein EGFP (mito-GFP) was targeted to the ubiquitously expressed Rosa26 locus, along with an upstream loxP-flanked termination signal, by CRISPR/Cas9 strategy. We have bought B6.129-Tg^(Pcp2–cre)2Mpin/J^ transgenic mice (named PCs-Cre; Strain number: 004146; RRID:IMSR_JAX:004146 Info; Common Name: L7Cre-2; The Jackson Laboratory), who express a cre gene inserted into exon 4 of a Pcp2 gene. Recombinase activity is observed in most Purkinje cells (PCs) and some retinal bipolar neurons and is first observed around postnatal day 6 and is fully established 2–3 weeks after birth. Pcp2-Cre mice from Jackson Laboratory (America, Stock No: 004146). Then we crossed the Mito-GFP mice with PCs-Cre mice, PCs-Mito-GFP mice were built in which the targeted LoxP allele can be mated to a Cre driver line to obtain cell-specific labeling of mitochondria. Figure 1A showed the germline excision of the termination signal produces the PCs-Mito-GFP line.

Figure 1B showed the PCR genotyping of the wildtype (on the left), Pcp2-Cre (the second line on the left), Pcp2-Mito-GFP (the second line on the right), and GFP-Mito tag^floxed^ strain (on the right), by using the three primers in schematic. From the results, we found that number 4 and 5 are the target mice, which were used in our research.

Figures 1C–F have indicated that in the cerebellum of PCs-Mito-GFP mice, all mitochondria within Purkinje cells expressed bright green fluorescence under a Zeiss confocal microscope equipped with Airyscan 2 system, while all other mitochondria all over the brain and cerebellum had no fluorescence.

The above results indicate that the present transgenic mice could be used for further observation.

### 3.2. Step 1: 2D images of MNs

According to 4-step process (Figure 2), we firstly observed the morphology of mitochondrial networks (MNs) in three subcellular units of Purkinje cells of axons, soma and dendrites.

**FIGURE 3:**
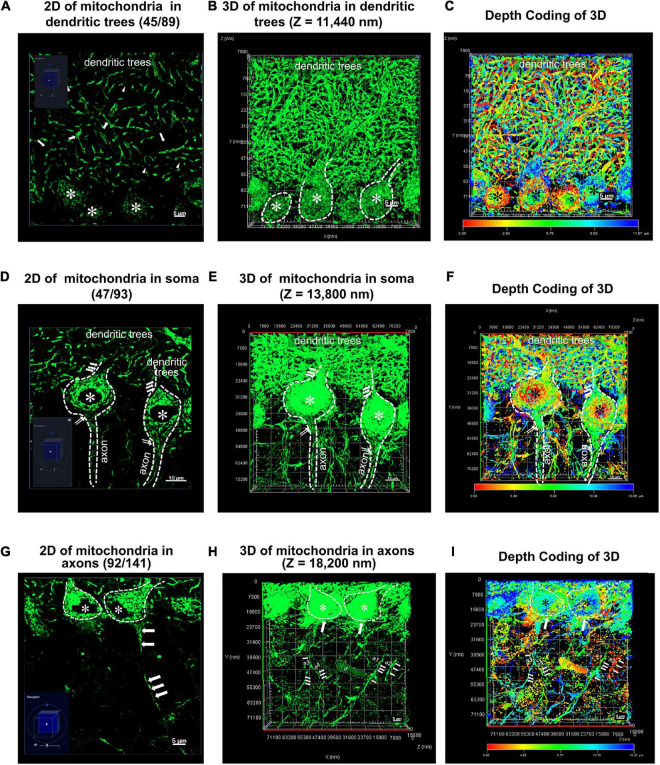
Two and three dimensional (2D and 3D) images acquisition of mitochondrial network within Purkinje cells from PCs-Mito-GFP mice. **(A,D,G)** A typical two dimensional (2D) image under light microscope (LM) for mitochondrial network within dentritic trees **(A)**, soma **(D)** and axons **(G)** of cerebellar Purkinje cells (PCs) from a PCs-Mito-GFP mouse. The navigation diagram in the bottom left corner indicates the exact position of image. Soma of PCs are indicated by asterisk (*) and one long axon indicated by a series of arrows. **(B,E,H)** A three dimensional (3D) image with z stack of 11,440 nm **(B)**, 13,800 nm **(E)** and 18,200 nm **(H)** is set up from 2D images of panels **(A,D,G)**, respectively. We find an unknown debris (d) in panel **(H)**. **(C,F,I)** A 3D image is set up under Depth Coding mode from 3D image in panels **(B,E,H)**. The Depth Coding diagram on the bottom indicates that the red dots lie on the superficial layer and the blue dots on the deep layer. The same debris (d) appearing in panel **(H)** is yellow color which suggests its location as bottom in the depth. Bars = 5 μm in panels **(A,G)**, and Bars = 10 μm in panel **(D)**.

Figure 3A showed there were 89 layers of typical 2D image under light microscope for MNs within the dendritic trees of Purkinje cells from a PCs-Mito-GFP mouse. We noticed that Purkinje cells’ soma were easy to be distinguished, however, it was hard to outline the complicated dendritic trees by using the green fluorescence of MNs. Moreover, the MNs in the dendritic trees appeared so complicated that: some of them looked like broken-line-shape with about 5–20 μm long (indicated by arrows), some of them shaped as bifurcated type (indicated by the dovetail arrows), and some of them shaped as short linear type (indicated by triangles).

Figure 3D showed there were 93 layers of typical 2D image under light microscope for MNs within the soma of Purkinje cells from a PCs-Mito-GFP mouse. The area of every somatic body was approximately 347.44 μm^2^, as well as their projecting thin axons and flourish incoming dendrites could be easily distinguished and outlined. The MNs in soma could not be depicted stereoscopically.

Figure 3G showed there were 141 layers of typical 2D image under light microscope for MNs within the axons of Purkinje cells from a PCs-Mito-GFP mouse. Less than 10 axons with the length of up to 30 μm could be distinguished (marked by white arrowheads). The MNs in axons could not be depicted stereoscopically.

### 3.3. Step 2: 3D images of MNs

Then after Z stacked processing in depth of approximately 20 μm for total more than 80 2D images in STEP 1, 3D reconstruction for MNs in Purkinje cells of PCs-Mito-GFP mice were made.

Figures 3B, E, H showed, in the second step, we acquired 3D images after Z stacked processing, in depth of 11.44 μm for total 89 images of MNs within the dendritic trees (Figure 3B), in depth of 13.8 μm for total 93 images of MNs within the soma (Figure 3E), as well as in depth of 18.2 μm for total 141 images of MNs within the axons (Figure 3H).

Figures 3C, F, I showed we further performed advanced processing according to Depth Coding of 3D within the dendritic trees (Figure 3C), the soma (Figure 3F), as well as the axons (Figure 3I). Red represented the bottom layer, and blue represented the top layer. At least we could distinguish the mitochondrial networks either in the superficial layer or in the deeper layer.

Thereafter the MNs within three subcellular units could be shown close to stereoscopically. Unfortunately, it was impossible to measure or compare MNs precisely.

### 3.4. Step 3: Primary immersive VR observation

Supplementary Movie 1 showed that, after advanced processing, we could freely track and observe MNs in Purkinje cells stereoscopically and precisely by using primary immersive VR. The girl postgraduate student on the left of the movie was the first author of the present manuscript, Hui Liu, who was wearing glasses on the eyes and a handle on two hands. On the right side of the screen, the merged 141 layers of light microscope images focusing on axons of Purkinje cells of PCs-Mito-GFP mice were generated. We could track and detect the green MNs which outlined liner axons and interwound with each other by using immersive primary VR technique. Dense green dots formed two kinds of structures, one was round soma located in the Purkinje cells layer (PCL), and another was liner axons located in the granular cells layer (GCL) of cerebellar cortex.

**FIGURE 4:**
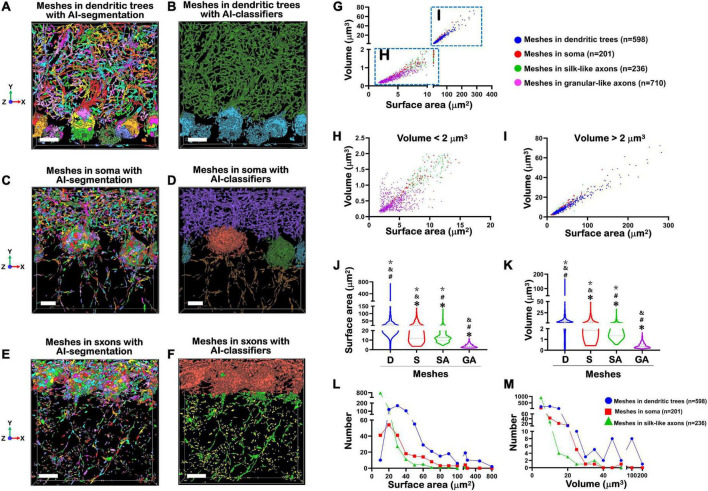
Treatment of MNs by AI-powered segmentation and classifiers. **(A)** Showed after Aivia’s AI-powered segmentation but without classifiers, the MNs in the dendritic trees of PCs were transformed to colorful meshes. These images were corresponding to Figures 3A–C. Difference of colors did not indicate the difference of size, but only represented the segmentation of different meshed. The total number of mitochondrial meshes was 598. Bar = 10 μm. **(B)** After treatment by Aivia’s AI-powered classifiers, all 598 mitochondrial meshes in the dendritic trees of PCs were given a dark green pseudo-color. Bar = 10 μm. **(C)** After Aivia’s AI-powered segmentation but without classifiers, the MNs in the soma of PCs were transformed to colorful meshes. These images were corresponding to Figures 2F, 3D. The total number of mitochondrial meshes was 201. Bar = 5 μm. **(D)** After treatment by Aivia’s AI-powered classifiers, all 201 mitochondrial meshes in the soma of PCs were given orange, or dark green, or lake color pseudo-color. Bar = 5 μm. **(E)** After Aivia’s AI-powered segmentation but without classifiers, the MNs in the axons of PCs were transformed to colorful meshes. These images were corresponding to Figures 3G–I. It should be indicated that the MNs in the axons could be divided into two types: one type was named as silk-like axons because the mitochondrial meshes connected to each other and formed a long liner line going through the GCL; another type was named as granular-like axons because the mitochondrial meshes distributed as an isolated island and they did not connect each other. The total number of silk-like mitochondrial meshes in axons was 236, while the total number of granular-like mitochondrial meshes in axons was 710. Bar = 10 μm. **(F)** After treatment by Aivia’s AI-powered classifiers, all 236 silk-like mitochondrial meshes in the axons were given green pseudo-color, while all 710 granular-like mitochondrial meshes in the axons were given yellow pseudo-color. Bar = 10 μm. **(G)** The relationship of surface area and volume of all 1,745 mitochondrial meshes, including in granular-silk axons (pink), in dendritic trees (blue), in soma (red) and in silk-like axons (green), showed near-linear correlations. **(H)** The relationship of surface area and volume of the smaller mitochondrial meshes with the volume less than 2 μm^3^. **(I)** The relationship of surface area and volume of the bigger mitochondrial meshes with the volume more than 2 μm^3^. **(J,K)** The comparison of the surface area (μm^2^) **(J)** and volume (μm^3^) **(K)** in dendritic trees (D), soma (S), silk-like axons (SA) and granular-like axons (GA). The values of mitochondrial meshes in four groups showed statistically significantly. *Compared to the number in dendritic trees group; ^#^compared to the number in soma group. ^&^Compared to the number in silk-like axons group. *Compared to the number in granular-like axons group. **(L,M)** The relationship between the number of mitochondrial meshes in dendritic trees (blue ball), or in soma (red square), or in silk-like axons (green triangle), and corresponding surface area **(L)** or volume **(M)**. Yellow part indicated the distribution of the most number of mitochondrial meshes. Statistical analysis was performed by Kruskal-Wallis test. The data are shown as the mean ± SD.

Supplementary Movie 2 showed the MNs in dendritic trees and all 89 images were corresponding to Figure 3A, Figure 4C. From the 1″ to 11″ of the movie, we could see dense green MNs in dendritic trees within the molecular layer (ML) of cerebellar cortex. From 12″ to 18″, or from 19″ to 23″, or from 24″ to 28″ of the movie, the dashed line outlined the somatic bodies of one Purkinje cell within PCL. We could see the full view of MNs of dendritic trees in ML. The bright green slender MNs connected each other and formed numberless irregular and shorter linear structures in a way similar to that in axons. But different from that in axons, the MNs in dendritic trees did not look like a string of knots one by one, but rather continuous. From 41″ to 58″, or 59″ ∼ 1′04″, or 1′10″ ∼ 1′12″, or 1′36″ ∼ 1′42″, or 1′43″ ∼ 1′48″, or 2′11″ ∼ 2′17″ of the movie, the dashed line outlined the short MNs of dendritic trees in ML. It should be indicated there were many MNs distributed in the dendrite shaft which were shown from 2′55″ to 3′05″ or from 3′08″ to 3′15″ of the movie.

Supplementary Movie 3 showed the MNs in Purkinje cells’ soma and all 93 images were corresponding to Figures 3D–F. From the 1″ to 6″ of the movie, we could see white line-outlined hexagonal-shaped MNs in soma within the PCL of cerebellar cortex and every unit of MNs shaped as pentagon. Every pentagon-shaped mitochondrial unit seemed to be in a 2D plane. By handling and rotating the VR, we further could find the hexagonal-shaped MNs in soma seemed to be in a 2D plane. At the 50″ of the movie, we could see 4 white line-outlined hexagonal-shaped MNs in soma within the PCL of cerebellar cortex, and every unit of MNs shaped as pentagon again. By handling and rotating the VR, we could find the hexagonal-shaped MNs in soma seemed to be in a 2D plane. In this movie, we could number no less than 20 such hexagonal-shaped MNs, which although located in different directions, surrounded around the empty core which should be nuclei of Purkinje cells.

Supplementary Movie 4 showed the MNs in axons and all 141 images were corresponding to Figures 3G–I (in which the 4 axons were marked as a1 – 4). From the 1″ to 22″ of the movie, the dashed line outlined axon 3. The appeared handle in the picture indicated the liner axons originating from the bottom of soma and sending through to the GCL layer. The total length was about 15 μm. From the 23″ to 33″ of the movie, the dashed line outlined axon 4. The appeared handle in the picture indicated the liner axons branched from the shaft of axon 3 and sending through to the GCL layer. From the 34″ to 46″ of the movie, the picture was dragged even closer to enlarge axons 3 and 4. From the 47″ to 1′05″ of the movie, we could see the axon 3 contained bright green mitochondria-like slender structures (i.e., mitochondrial mesh described in the following parts), in addition, these structures connected up and down, which made the MNs in axons of PCs look like a string of knots one by one. From the 1′06″ to 1′25″ of the movie, we could see the axon 1 outlined by dashed white line. The appeared handle in the picture retrogradely indicated the liner axons sending through to the GCL layer and originated from the bottom of the Purkinje cells’ soma. The bright green slender MNs connected up and down and formed a string of green knots. From the 1′26″ to the end of the movie, we could see the shorter axon 2 outlined by dashed white line. The bright green slender MNs connected up and down and formed a string of green knots.

### 3.5. Aivia’s AI-powered segmentation and classifiers in front of step 4

Then by use of Aivia’s AI-powered segmentation, we for the first time found that neuronal MNs were composed of the fundamental units, named mitochondrial meshes.

Figure 4A showed that, after Aivia’s AI-powered segmentation, the MNs in the dendritic trees of Purkinje cells were transformed to colorful meshes. These images were corresponding to Figures 3A–C. The difference of colors did not indicate the difference of size, but only represented the segmentation of different meshes. The total number of mitochondrial meshes was 598. Accordingly, after Aivia’s AI-powered segmentation, the MNs in the soma of PCs were transformed to colorful meshes.

Figure 4C showed that, after Aivia’s AI-powered segmentation, the MNs in the soma of Purkinje cells were transformed to colorful meshes. These images were corresponding to Figures 3D–F. The total number of mitochondrial meshes was 201.

Figure 4E showed that, after Aivia’s AI-powered segmentation, the MNs in the axons of PCs were transformed to colorful meshes. These images were corresponding to Figures 3G–I. It should be indicated that the MNs in the axons could be divided into two types: one type was named as silk-like axons because the mitochondrial meshes connected to each other and formed a long liner line going through the GCL; another type was named as granular-like axons because the mitochondrial meshes distributed as an isolated island and they did not connect each other. The total number of silk-like mitochondrial meshes in axons was 236, while the total number of granular-like mitochondrial meshes in axons was 710.

Then by use of Aivia’s AI-powered classifiers, we for the first time calculated the parameters of surface area and volume of every mitochondrial mesh. Table 1 showed the results of analysis of mitochondrial meshes in different subcellular units of Purkinje cells in PCs-Mito-GFP mice.

**TABLE 1:** Analysis of mitochondrial meshes in different subcellular units of PC in PCs-Mito-GFP mice.

Location	Number	Surface area (μm^2^)			Volume (μm^3^)		
		**Max**	**Min**	**Average**	**Max**	**Min**	**Average**
Dendritic trees	598	742.98	0.00014	45.30 ± 53.21^#&^*	167.20	8.940e-005	9.97 ± 12.34^#&^*
Soma	201	136.50	3.53	27.81 ± 22.22*^&^*	47.95	0.42	6.26 ± 6.46*^&^*
Silk-like axons	236	130.20	4.57	17.50 ± 15.19*^#^*	32.77	0.53	3.52 ± 4.29*^#^*
Granular-like axons	710	11.89	1.70	3.99 ± 1.82*^#&^	1.63	0.11	0.43 ± 0.25*^#&^

*Significant comparison between the number in other three groups and the number in dendritic trees group.

^#^Significant comparison between the number in other three groups and the number in soma group.

^&^Significant comparison between the number in other three groups and the number in silk-like axons group.

*Significant comparison between the number in other three groups and the number in granular-like axons group.

Figure 4B showed that after treatment by Aivia’s AI-powered classifiers, all 598 mitochondrial meshes in the dendritic trees of Purkinje cells were given a dark green pseudo-color. At the same time, the mitochondrial meshes in the soma of Purkinje cells were given a lake blue pseudo-color.

Figure 4D showed that after treatment by Aivia’s AI-powered classifiers, all 201 mitochondrial meshes in the soma of were given orange, or dark green, or lake color pseudo-color.

Figure 4F showed that after treatment by Aivia’s AI-powered classifiers, all 236 silk-like mitochondrial meshes in the axons were given green pseudo-color, while all 710 granular-like mitochondrial meshes in the axons were given yellow pseudo-color.

Figure 4G showed the distribution trend of surface area and volume of all 1,745 mitochondrial meshes. It could be found that the meshes in granular-silk axons (pink) were concentrated within the lower left corner of the statistical graph, which meant they had the smallest size (Figure 4H). On the contrary, the meshes in dendritic trees (blue) were concentrated within the upper right corner of the statistical graph which meant they had the biggest size (Figure 4I). And the meshes in soma (red) and silk-like axons (green) were distributed in the middle of the graph.

Figure 4H showed when we calculated the smaller meshes with the volume less than 2 μm^3^, most of these meshes were pink mitochondrial meshes within the granular-silk axons. Figure 4I showed when we calculated the biggest meshes with the volume more than 2 μm^3^, most of these meshes were blue mitochondrial meshes within the dendritic trees.

Figure 4J showed the surface area (μm^2^) was the biggest in dendritic trees (45.30 ± 53.21), the smallest in granular-like axons (3.99 ± 1.82), and in the middle in soma (27.81 ± 22.22) and silk-like axons (17.50 ± 15.19). The values of mitochondrial meshes in four groups showed statistically significantly (*P* < 0.001).

Figure 4K showed the volume (μm^3^) was also the biggest in dendritic trees (9.97 ± 12.34), the smallest in granular-like axons (0.43 ± 0.25), and in the middle in soma (6.26 ± 6.46) and silk-like axons (3.52 ± 4.29). The values of mitochondrial meshes in four groups showed statistically significantly.

Figure 4L showed the relationship between the number of mitochondrial meshes and corresponding surface area. We could find the most number of mitochondrial meshes whether in dendritic trees (blue ball), or in soma (red square), or in silk-like axons (green triangle) had the surface area of 20–30 μm^2^ (yellow part).

Figure 4M showed the relationship between the number of mitochondrial meshes and corresponding volume. We could also find the most number of mitochondrial meshes whether in dendritic trees (blue ball), or in soma (red square), or in silk-like axons (green triangle) had the volume of less than 10 μm^3^ (yellow part).

### 3.6. Step 4: Secondary immersive VR observation

Supplementary Movie 5 showed the secondary immersive VR with Aivia’s AI-powered segmentation but without classifiers of meshed MNs in dendritic trees of PCs. All 89 images were corresponding to Figures 2A–C. The left side of the movie showed the primary VR; the right side showed the secondary VR after Aivia’s AI-powered segmentation but without classifiers, which were corresponding to Figure 4A. It could be achieved to drag, enlarge and even step into the meshed MNs. We could see different colors that did not indicate the size of mitochondrial meshes within the dendritic trees of PCs, but represented the segmentation of meshes. We observed the meshes connected head to end along the long axis, so formed long chains.

Supplementary Movie 6 showed the secondary immersive VR with both Aivia’s AI-powered segmentation and classifiers of meshed MNs in dendritic trees of PCs, which were corresponding to Supplementary Movie 5. All mitochondrial meshes in the dendritic trees of PCs were given a dark green pseudo-color, and the mitochondrial meshes in the soma of PCs were given a lake blue pseudo-color. We further calculated the parameters of surface area and volume of every mitochondrial mesh by using this data.

Supplementary Movie 7 showed the secondary immersive VR with Aivia’s AI-powered segmentation but without classifiers of meshed MNs in soma of PCs. All 93 images were corresponding to Figures 3D–F. It showed the secondary VR after Aivia’s AI-powered segmentation but without classifiers, which were corresponding to Figure 4C. We observed the mitochondrial meshes within the somatic bodies connected into pieces and formed a sponge-like structure. It was interesting to find that these meshes looked like a jigsaw puzzle due to their different colors and shapes. Each mitochondrial mesh could be seen as a fundamental unit which owned a main body and sent to no less than 5 short branches to different directions for further connecting.

Supplementary Movie 8 showed the secondary immersive VR with both Aivia’s AI-powered segmentation and classifiers of meshed MNs in soma of PCs, which were corresponding to Supplementary Movie 7. All mitochondrial meshes in the dendritic trees of PCs were given a purple pseudo-color, and the mitochondrial meshes in the soma of PCs were given a lake blue or deep red dark green pseudo-color.

Supplementary Movie 9 showed the secondary immersive VR with Aivia’s AI-powered segmentation but without classifiers of meshed MNs in axons of PCs. All 141 images were corresponding to Figures 3E–H. It showed the secondary VR after Aivia’s AI-powered segmentation but without classifiers, which were corresponding to Figure 4E. It showed the mitochondrial meshes in the axons could be divided into two types: one type was named as silk-like axons because the mitochondrial meshes connected to each other just like them in dendritic trees; another type was named as granular-like axons because they were distributed as an isolated island.

Supplementary Movie 10 showed the secondary immersive VR with both Aivia’s AI-powered segmentation and classifiers of meshed MNs in axons of PCs, which were corresponding to Supplementary Movie 9. All mitochondrial meshes in the soma of PCs were given a deep red pseudo-color, and the silk-like mitochondrial meshes in the axons were given a dark green pseudo-color, and the granular-like mitochondrial meshes in the axons were given a yellow pseudo-color.

We also showed 50 pieces of VR images of mitochondrial meshes within each group of dendritic trees (Supplementary Figure 2), soma (Supplementary Figure 3) and axons (Supplementary Figure 4), combined with the corresponding 3D ply files in Supplementary Figures 2–4.

Based on these results, we put forward the hypothesis that neuronal MNs were composed by regular meshes whose surface area and volume were the biggest in dendritic trees, the smallest in axons, and moderate in soma.

### 3.7. Confirmation of the mitochondrial mesh hypothesis by using the model of simulating long-term pilot flight

**FIGURE 5:**
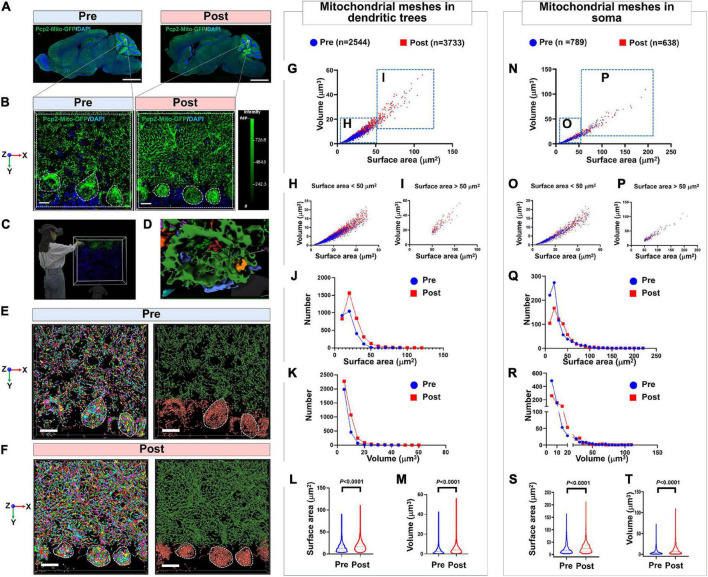
Confirmation of the mitochondrial mesh hypothesis by using the model of simulating long-term pilot flight. **(A)** Panoramic view the whole brain of PCs-Mito-GFP mice before or after the treatment of overweight centrifugal force. Bar = 1 mm. **(B)** Magnification of PCs from panel **(A)**. Bar = 5 μm. **(C)** 3D reconstruction view is observed by immersive VR. **(D)** The magnification showing the meshes in soma of PC. Bar = 20 μm. **(E,F)** The quantity of mitochondria in dendritic trees and soma from PCs-Mito-GFP mice increases sharply after treatment with overweight centrifugal force. Bar = 5 μm. **(G)** Scatter diagram of mitochondrial mesh in dendritic trees of PCs. **(H,I)** Scatter diagram of mitochondrial mesh, whose volume is smaller than 50 μm^3^ **(H)** or larger than 50 μm^3^ **(I)**, in dendritic trees of PCs. **(J,K)** Frequent distribution showing the surface area **(J)** and volume **(K)** of mitochondrial mesh in dendritic trees of PCs. **(L,M)** Quantification showing the surface area **(L)** and volume **(M)** of mitochondrial mesh in dendritic trees of PCs. The surface area and volume of mitochondrial mesh in dendritic trees of PCs increased remarkably after treatment with overweight centrifugal force. **(N)** Scatter diagram of mitochondrial mesh in soma of PCs. **(O,P)** Scatter diagram of mitochondrial mesh, whose volume is smaller than 50 μm^3^ **(O)** or larger than 50 μm^3^ **(P)**, in soma of PCs. **(Q,R)** Frequent distribution showing the surface area **(Q)** and volume **(R)** of mitochondrial mesh in soma of PCs. **(S,T)** Quantification showing the surface area **(S)** and volume **(T)** of mitochondrial mesh in soma of PCs. The surface area and volume of mitochondrial mesh in soma of PCs increased remarkably after treatment with overweight centrifugal force. Mann-Whitney *U*-test was used for statistical analysis. The data are shown as the mean ± SD.

It has been shown that after long-term professional training and flight practice, pilots would acquire more skilled driving skills and a much more precise sense of direction (Jillings et al., 2020). The underlying mechanisms are closely related to neuroplasticity of the adaptive structural and functional changes occurring in the brain, especially in the cerebellum, which is able to deal with a microgravity environment changes. However, this mechanism is still unclear. In the present study, we designed the small animal overweight simulated centrifuge machine and trained the PCs-Mito-GFP mice in these machines. We compared the surface area and the volume of the meshes of MNs in PCs in pre- and post-treatment group. Figures 5A–F showed we used 4-step process described above composed of 2D and 3D observation as well as primary and secondary AI-powered VR to make the comparison. Table 2 showed the analysis results. Figures 5G–M showed the surface area of mitochondrial meshes within the dendritic tress increased significantly in the post-treatment group (19.17 ± 12.48 μm^2^) when compared with that in the pre-treatment group (14.93 ± 9.60 μm^2^) (*P* < 0.001); similarly, the volume of mitochondrial meshes within the dendritic tress increased significantly in the post-treatment group (5.41 ± 5.15 μm^3^) when compared with that in the pre-treatment group (3.52 ± 3.30 μm^3^) (*P* < 0.001). Figures 5N–T showed the surface area of mitochondrial meshes within the soma increased significantly in the post-treatment group (29.55 ± 24.39 μm^2^) when compared with that in the pre-treatment group (23.20 ± 21.78 μm^2^) (*P* < 0.001); similarly, the volume of mitochondrial meshes within the soma increased significantly in the post-treatment group (9.86 ± 11.71 μm^3^) when compared with that in the pre-treatment group (7.16 ± 9.91 μm^3^) (*P* < 0.001). Thereafter we indicated the meshed neuronal MNs might be the fundamental unit best for real-time fluctuated and complex mitochondria function.

**TABLE 2:** Analysis of mitochondrial meshes in different subcellular units before or after simulated long-term pilot flight within PCs in PCs-Mito-GFP mice.

Pre-or post-treatment	Location	Number	Surface area (μm^2^)			Volume (μm^3^)		
			**Max**	**Min**	**Average**	**Max**	**Min**	**Average**
Pre-	Dendritic trees	2,544	90.91	3.13	14.93 ± 9.60*	42.51	0.20	3.52 ± 3.30*
	Soma	789	163.30	3.43	23.20 ± 21.78*	72.62	0.33	7.16 ± 9.91*
Post-	Dendritic trees	3,773	110.80	3.00	19.17 ± 12.48*	56.10	0.28	5.41 ± 5.15*
	Soma	638	211.00	3.44	29.55 ± 24.39*	109.00	0.43	9.86 ± 11.71*

*Significant comparison between the number in pre- and post-simulated long-term pilot flight.

## 4. Discussion

In this report, we developed a framework to capture and analyze the neuronal mitochondrial networks by using 4-step composed of 2D and 3D observation, primary and secondary virtual reality (VR) analysis, with the help of artificial intelligence (AI)-powered Aivia segmentation and classifiers. The 4-step process allowed us to propose that the neuronal mitochondrial networks might be composed of regular mitochondrial meshes, and more importantly, provide the detailed quantitative measurement of the two important parameters of the surface area and the volume of neuronal mitochondrial meshes, by using the generated PCs-Mito-GFP mice in which the mitochondria specific within the cerebellar Purkinje cells are labeled by GFP. The results showed that both the surface area and the volume of mitochondrial meshes in Purkinje cells were the biggest in dendritic trees, the smallest in granular-like axons, and moderate in soma and silk-like axons. Moreover, both the surface area and the volume of mitochondrial meshes in dendritic trees and soma within the Purkinje cells in PCs-Mito-GFP mice, who were trained by the simulating long-term pilot flight concentrating, showed significantly increased. Hence we propose that the AI-powered segmentation and classifiers, combined with the immersive VR reconstruction could resolve the complicated neuronal mitochondrial networks into discrete and quantifiable mitochondrial meshes, so as to achieve the detailed morphological analysis of neuronal mitochondrial networks.

The morphology of mitochondrial networks (MNs), a term that encompasses the branched, reticular structure of fused mitochondria as well as the separate, punctate individual organelles within a eukaryotic cell. Over the past decade, the significance of the mitochondrial network has been increasingly appreciated, motivating the development of various approaches to analyze it. Protocols enabling imaging of mitochondrial morphological network are currently available for both wide-field epifluorescence microscopy and high-resolution laser scanning confocal microscopy (Mitra and Lippincott-Schwartz, 2010; Chevrollier et al., 2012; Nikolaisen et al., 2014; Ekanayake et al., 2015). A custom-built super-resolution microscope has also been used to image submitochondrial distribution of voltage-dependent anion channel isoforms, but only in fixed cells (Neumann et al., 2010). Airyscan super-resolution microscopy has been used for studying mitochondrial morphology and dynamics in living tumor cells through analyzing mitochondrial number and its volume (Kolossov et al., 2018). Valente et al. (2017) have reported that the MiNA toolset, making use of existing ImageJ plug-ins, allows for semi-automated analysis of mitochondrial networks in cultured mammalian cells. MiNA converts the images to binary and produces a morphological skeleton for calculating nine parameters to quantitatively capture the morphology of the mitochondrial network (Valente et al., 2017). Harwig et al. (2018) have reported that the MitoGraph, an opensource image analysis platform for measuring mitochondrial morphology based on imageJ post-image processing, could successfully differentiate between distinct mitochondrial morphologies that ranged from entirely fragmented to hyper-elongated with use of confocal imaging. Rohani et al. (2020) have reported a collection of independent tools, Mito Hacker, which aims to process cellular mitochondrial images at different levels of specificity; 2d Multi-cell RGB images, 2d single-cell RGB images, and 2d binary single-cell images. Mito Hacker is developed in python 3.7 and uses 14 different python libraries across different tools to analyze the images. Baptista and De Bacco (2021) have proposed a principled optimization frame model that outputs a network representing the topological structure of mitochondrial network contained in the 2D confocal image. Ilamathi et al. (2021) have developed Mitomate tracker, an algorithm that takes advantage of mitochondrial network quantification tool Momito, to which we combined two other tools: the ImageJ plugin, Trackmate which allows nucleoid identification, and the R package, spatstat which calculates point pattern distributions (Ouellet et al., 2017; Tinevez et al., 2017). Fogo et al. (2021) have developed a semi-automated image analysis pipeline for the quantitation of mitochondrial morphology utilizing immunolabeling images as well as serial block-face scanning electron microscopy. The above methods are quantified by using Open Source software, imageJ or manual operation. The main quantitative indicators are the number, area, volume, and length of mitochondria. At the same time, the observation of mitochondrial morphology by the above methods is only limited to 2D plane, which makes the observation field limited.

Our research team has used VR technology for the first time to observe mitochondria in PCs. The present AiviaVR offers an immersive, intuitive, and realistic experience for exploring mitochondrial network imaging data, where real and virtual contents were synthetically put together to demonstrate the user experience of AiviaVR. While VR has not been widely used in neuroscience, it is useful for biological problems, especially due to the intrinsic multi-dimensional nature of many biological datasets, and has the potential to be integrated as the next standard protocol. AiviaVR is among the first demonstration of such utility with great potential. While immersive VR visualization of biological surface objects and sometimes also imaging data were shown in applications such as biological education and data analyses, there is little existing work on developing open-source VR software packages for very complicated and teravoxel-scale imaging datasets such as the whole-brain imagery as we have introduced here. We expect that AiviaVR can also be used to analyze other massive-scale datasets, especially changes of mitochondrial network under multiple stresses. We chose to focus on applying AiviaVR to the single-neuron reconstruction of mitochondrial network for two major reasons. First, currently no other alternative tools are able to reconstruct the fine, complicated mitochondrial network unambiguously in this way. Second, there has been little previous work on streamlining the large-scale data production of the complete mitochondrial network at single-neuron level at high precision and scale. We have uploaded all of these AiviaVR databases on our web of In order to provide an effective database for more mitochondrial researchers.

It is the first time to make use of artificial intelligence (AI)-powered Aivia segmentation an classifiers to study the complicated neuronal mitochondrial networks. In front of step 4, the segmentation and classifiers have been performed by using Aivia, and then the continuous matrix lumen shaped mitochondria networks could be converted to discrete neuronal mitochondrial meshes. Aivia uses an artificial intelligence (AI)-based software architecture to build a complete platform for two-dimensional to five-dimensional image visualization, analysis, and data interpretation that reliably processes and reconstructs highly complex images in just a few minutes. AI-powered segmentation is based on different convolutional neural network architectures to process images. And AI-powered classification, employing object classifier, is in terms of random forest. The Aivia’s AI is characterized by enabling complex, difficult and time-consuming image processing to be completed quickly, objectively and repeatably and efficiently, even when the analyst does not have the relevant expertise. AI is able to reliably process and reconstruct highly complex images in just a few minutes. Thus, reliable and repeatable segmentation results are generated to effectively and quickly realize 2D and 3D image visualization and analysis. In the present research, Aivia’s use of artificial intelligence will greatly improve our efficiency in quantifying mitochondria.

Although mitochondria are often depicted as discrete organelles, they actually form a highly dynamic and interconnected networks that undergoes continuous remodeling through rounds of organelle fusion and fission (Manfredi and Beal, 2007). Despite the mounting evidence that mitochondrial networks or dynamic is necessary in neurons, it has been unclear as to what exact shape they are. To address such problem, previous researches have generated a set of analysis systems allowing an unprecedented characterization of the morphological phenotypes associated with the mitochondrial network (Dempsey, 1956; Herndon, 1963; Landis, 1973; Martone et al., 1993; Pham et al., 2012). Herndon (1963) has described in 1963 that in the low power electron microscopic image of a Purkinje cell, mitochondria can be seen scattered throughout the cytoplasm along with numerous elements of granular endoplasmic reticulum. Several mitochondria in close association with subsurface cisterns are also seen (Herndon, 1963). Mitochondria in PCs are generally sausage-shaped and in most instances the cristae run at right angles to the long axis. Occasional irregular and branched forms are seen. The mitochondria in the smaller dendritic branches, on the other hand, tend to be more elongated and their cristae usually run parallel to the long axis of the mitochondrion. These elongated mitochondria are often helpful in identifying the smaller dendritic branches of the PCs. Our present results about the appearance of the profiles of mitochondrial networks in the axons and dendritic trees of PCs are consistent with previous report by Landis S C. that they are long and sinuous cylinders (Landis, 1973). And the shapes of mitochondrial profiles in the perikarya (soma) of normal PCs range from long and ellipsoidal to small and circular with an average diameter of 0.42 μm. Landis (1973) has described the ultrastructural characteristic of the mitochondria of cerebellar PCs. The interpretation most consistent with the appearance of the profiles is that the mitochondria are long, sinuous cylinders randomly oriented within the cell body. Ten such mitochondria in the primary dendrite of a 15-day normal mouse had an average length of 3.96 μm and an average width of 0.50 μm. In the present immersive AiviaVR study, the mitochondria in dendritic trees are cylinders, each one has an approximate volume of 0.78 μm^3^. Pham et al. (2012) have generated the mouse line of photo-activatable mitochondria (PhAM). In the *PhAM*^floxed^ line, a mitochondrial localized version of the photo-convertible fluorescent protein Dendra2 (mito-Dendra2) is targeted to the ubiquitously expressed Rosa26 locus, along with an upstream loxP-flanked termination signal. Then they crossed PhAMfloxed mice with the Pcp2-Cre line, which drives Cre expression in Purkinje cells (PCs) of the cerebellum.

But there are some limitations here. One is the lack of collaborative interaction, so in fact, the description is still subjective and cannot be quantified. Second, light microscope observation cannot avoid the false results due to light-induced diffraction. Third, the combination of light microscope and electron microscope should be more reliable to reveal the mitochondrial network in the future.

## 5. Conclusion

The present study created a four-step observational system of 2D/3D/primary VR/secondary VR to provide immersive evaluation of mitochondrial networks. The results of knot-like connected mitochondrial network within the axons and the dendritic trees, as well as the hexagonal two-dimensional unit for mitochondrial network within the soma, implies the further great effort should be addressed to elucidate the mitochondrial network. Future issues contain: 1. How does the mitochondrial network respond to multiple stresses? 2. How are different shapes of mitochondrial network form? 3. How does the mitochondrial network change in the absence of important mitochondrial dynamic factors, including fission factors of Drp1, Fis1, and fusion factors of Mfn1, Mfn2…?

## Data availability statement

The original contributions presented in this study are included in the article/Supplementary material, further inquiries can be directed to the corresponding authors.

## Ethics statement

The animal study was reviewed and approved by the Animal Care and Use Committee of the Air Force Military Medical University.

## Author contributions

Y-YW, Y-LY, FT, and LW designed the experiments. S-JL, HL, and F-FW conducted the experiments. HL, D-YF, and SZ conducted the part of AI-classification and VR-observation. S-JL, HL, and JZ analyzed the data and drew the figures. Y-YW, S-JL, and HL wrote the manuscript. All authors read and approved the final manuscript.

## Footnotes

1
http://cran.r-project.org/web/packages/party/

2
http://mouse.brain-map.org/static/atlas
