# Supplementary material for: Meshed neuronal mitochondrial networks empowered by AI-powered classifiers and immersive VR reconstruction
Source: Front Neurosci. 2023 Feb 2;17:1059965. doi: 10.3389/fnins.2023.1059965 (PMC9932543; doi:10.3389/fnins.2023.1059965)
Supplement: Supplementary file 1 [file Presentation_1.pdf]

Supplementary files

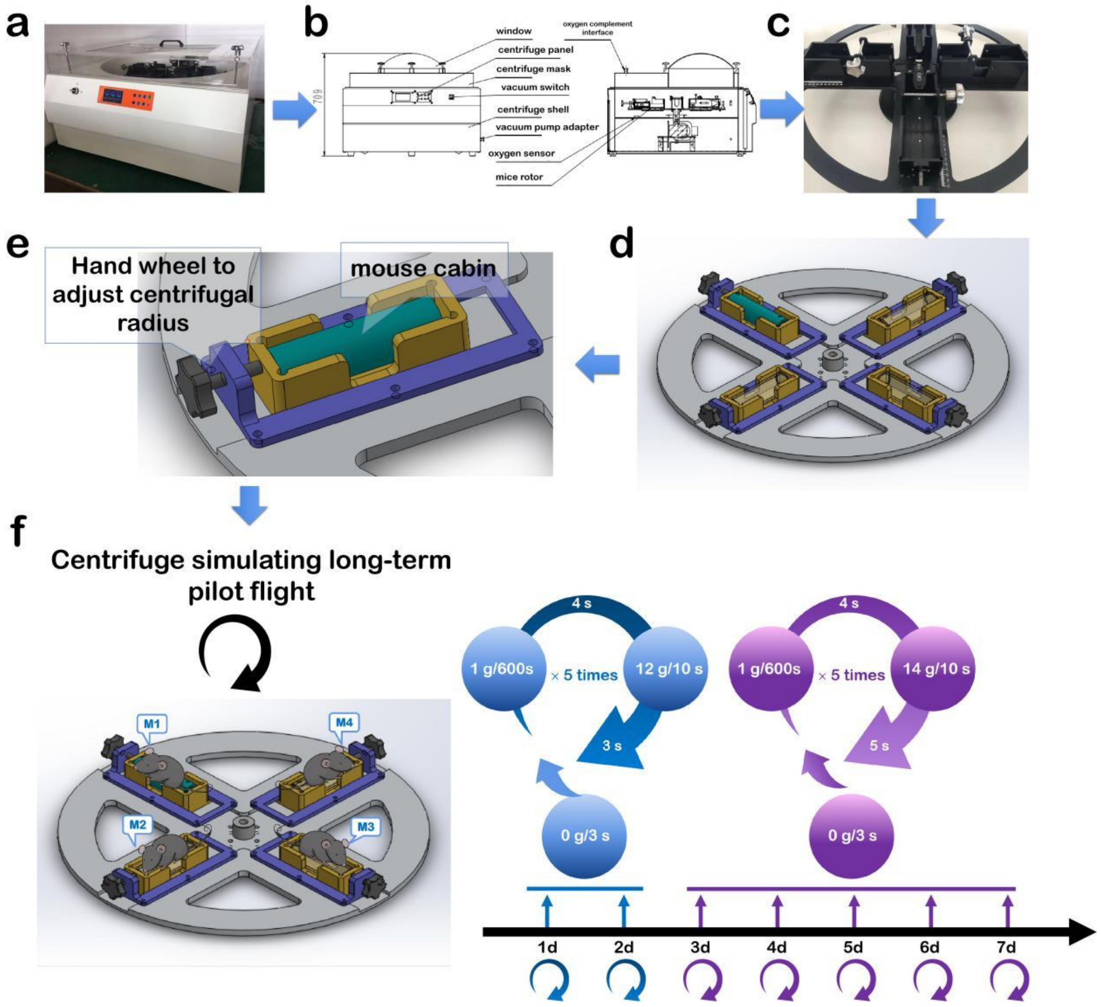

Supplementary Fig. 1. Apparatus and procedures of centrifuge simulating long-term pilot flight.

(a) The external view of centrifuge machine simulating long-term pilot flight.

(b) Schematic design of centrifuge machine simulating long-term pilot flight. The machine had 5 technical parameters: a, containing 4 mice cabins with 20 cm of centrifugation radius; b, 1~10 s duration for centrifugal gravity acceleration from 0 g to 2~20 g; c, the centrifuge can be kept stable to 1 g before and after starting the speed up; d, the vacuum pressure value ranged from 0 Kpa ~ 100 Kpa to simulate the plateau environment; e, oxygen concentration in the cavity can be detected and adjuested.

(c) The internal view of centrifuge machine simulating long-term pilot flight.

(d) Schematic design of the tray containing four mouse cabins.

(e) Schematic design of the mouse cabin apparatus with hand wheel.

(f) Centrifuge protocol. In the first 2 days, the centrifugal force firstly increased from 0 g to 1 g in 3 seconds and lasted for 10 minutes at 1 g, then the centrifugal force increased from 1 g to 12 g in 4 seconds and lasted for 10 seconds at 12 g which repeated five times. In the following five days, the centrifugal force firstly increased from 0 g to 1 g in 3 seconds and lasted for 10 minutes at 1 g, then the centrifugal force increased from 1 g to 14 g in 4 seconds and lasted for 10 seconds which repeated five times.

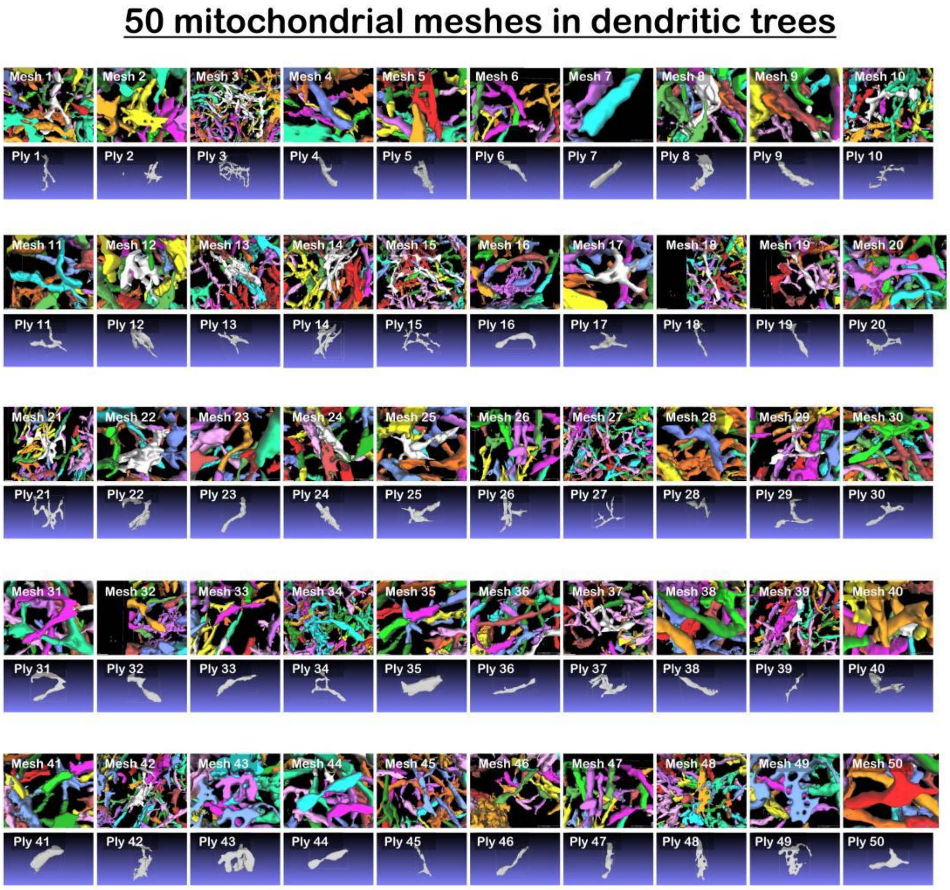

Supplementary Fig. 2. 50 pieces of VR images of mitochondrial meshes (upper rows) within dendritic trees, combined with the corresponding 3D ply files (lower rows).

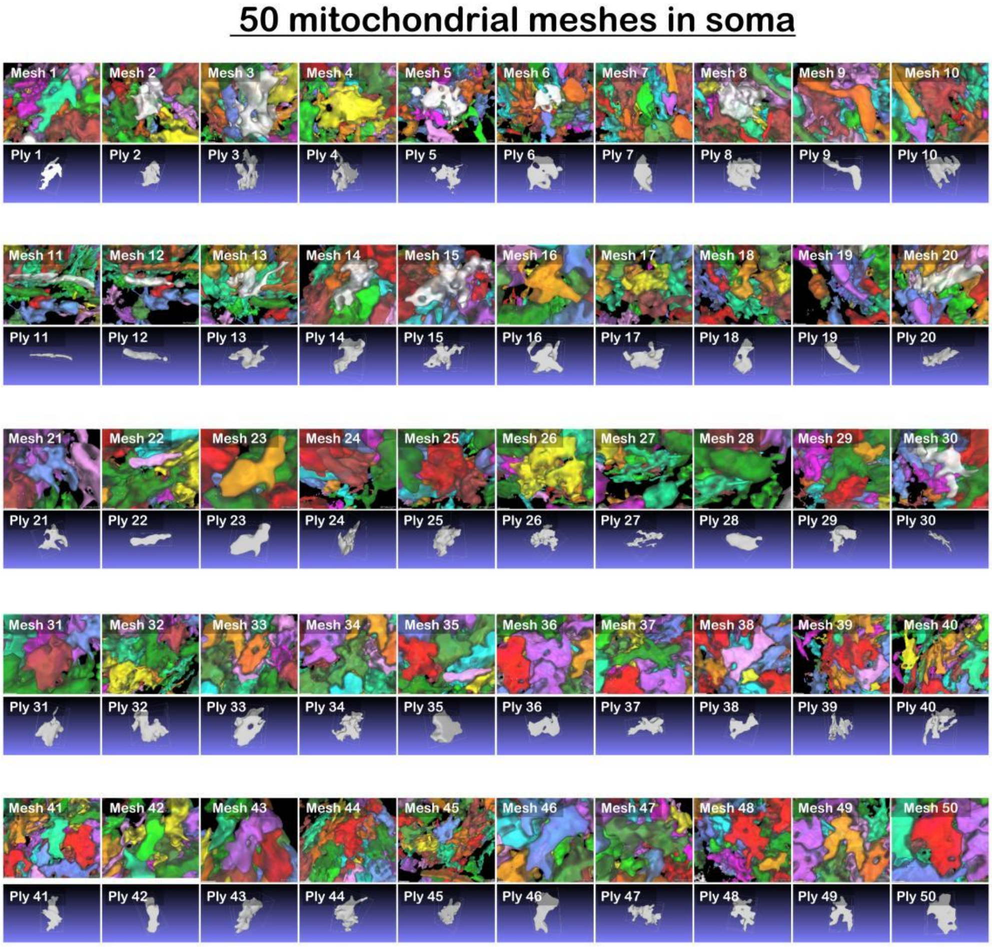

Supplementary Fig. 3. 50 pieces of VR images of mitochondrial meshes (upper rows) within soma, combined with the corresponding 3D ply files (lower rows).

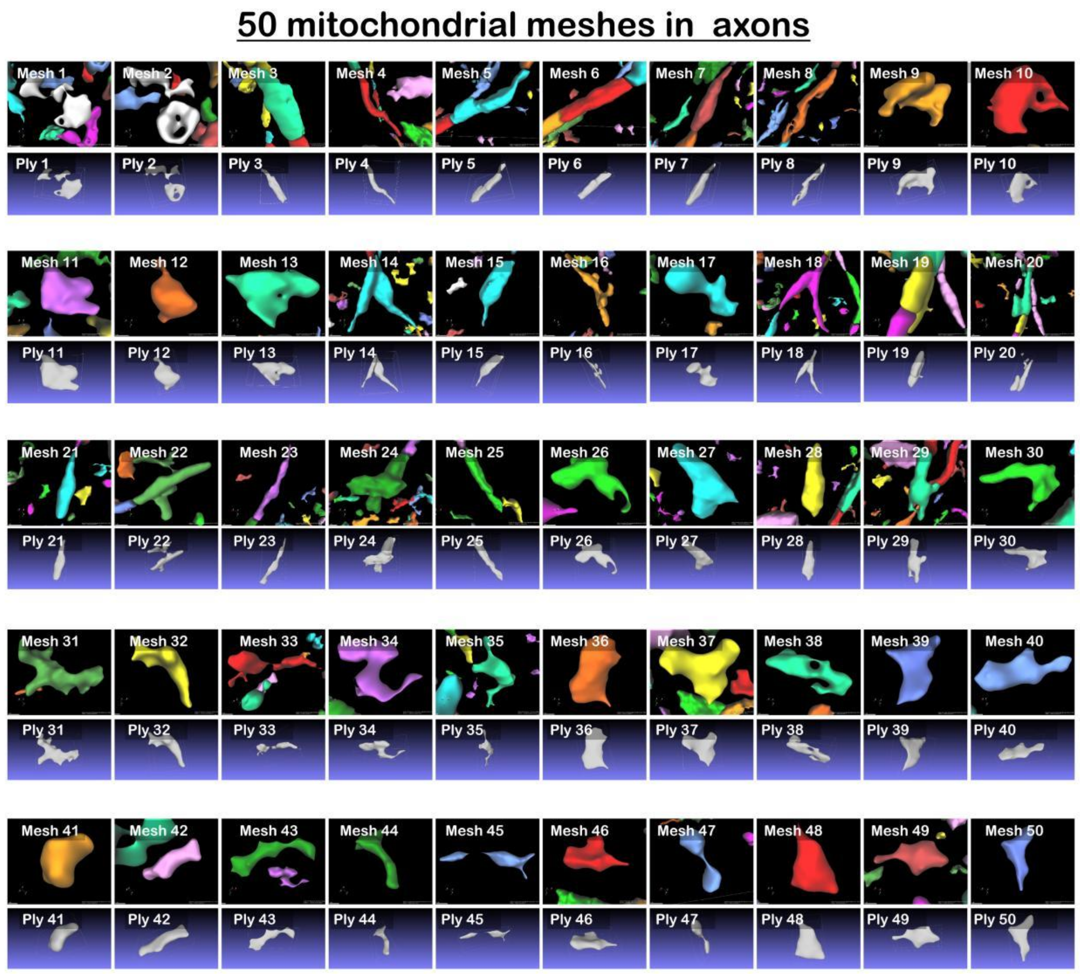

Supplementary Fig. 4. 50 pieces of VR images of mitochondrial meshes (upper rows) within axons, combined with the corresponding 3D ply files (lower rows).
