# Supplementary material for: Meshed neuronal mitochondrial networks empowered by AI-powered classifiers and immersive VR reconstruction
Source: Front Neurosci. 2023 Feb 2;17:1059965. doi: 10.3389/fnins.2023.1059965 (PMC9932543; doi:10.3389/fnins.2023.1059965)
Supplement: Supplementary file 2 [file Presentation_2.pdf]

Movie 1. Primary immersive VR of MNs.

The girl postgraduate student on the left of the movie was the first author of the present manuscript, Hui Liu, who was wearing glasses on the eyes and a handle on two hands. On the right side of the screen, the merged 141 layers of LM images focusing on axons of PCs of PCs-Mito-GFP mice were generated. We could track and detect the green MNs which outlined liner axons and interwound with each other by using immersive primary VR technique. Dense green dots formed two kinds of structures, one was round soma located in the Purkinje cells layer (PCL), and another was liner axons located in the granular cells layer (GCL) of cerebellar cortex. Blue dots of DAPI-marked nucleis could indicate the GCL. By using VR, we could flip freely MNs for immersive all-round observation. The movie was 3 minutes long.

Movie 2. Primary immersive VR of MNs in dendritic trees of PCs.

All 89 images were corresponding to Figs. 3A-3C.

From the 1" to 11" of the movie, we could see dense green MNs in dendritic trees within the molecular layer (ML) of cerebellar cortex.

From the 12" to 18" of the movie, the dashed line outlined one somatic body of one PC within PCL.

From the 19" to 23" of the movie, the dashed line outlined another somatic body of one PC within PCL.

From the 24" to 28" of the movie, the dashed line outlined the 3rd somatic body of one PC within PCL.

From the 29" to 40" of the movie, we could see the full view of MNs of dendritic trees in ML. The bright green slender MNs connected each other and formed numberless irregular and shorter linear structures in the way similar that in axons. But different from that in axons, the MNs in dendritic trees did not look like a string of knots one by one, but rather continuous,.

From the 41" to 58" of the movie, the dashed line outlined the short-rod-like MNs of dendritic trees in ML. Its length was about 5 μm.

From the 59" to 1'04" of the movie, the dashed line outlined another short-rod-like MNs of dendritic trees. Its length was about 3 μm.

From the 1'10" to 1'12" of the movie, the dashed line outlined DAPI-marked nuclei in ML.

From the 1'36" to 1'42" of the movie, the dashed line outlined several short-rod-like MNs of dendritic trees in ML.

From the 1'43" to 1'48" of the movie, the dashed line outlined a circle-like MNs of dendritic trees in ML.

From the 1'50" to 2'06" of the movie, the dashed line outlined a short-rod-like MNs of dendritic trees in ML. Its length was about 8 μm.

From the 2'11" to 2'17" of the movie, the dashed line outlined a circle-like MNs of dendritic trees in ML.

From the 2'55" to 3'05" of the movie, there appeared a round dot with the white edges of the red heart, and the dot moved and its pathways graved a profile of a dendritic shaft which returned to the somatic body and moved through the whole ML.There were many MNs distributed in this dendrite shaft.

From the 3'08" to 3'15" of the movie, there appeared another mixture of MNs describing a dendrite shaft outlined by white dotted line.

From the 3'16" to the end of the movie, DAPI-marked nuclei could distinguish PCL of cerebellar cortex.

Total duration of the movie was 3'33".

Movie 3. Primary immersive VR of MNs in soma of PCs.

All 93 images were corresponding to Figs. 3D-3F.

From the 1" to 6" of the movie, we could see white line-outlined hexagonal-shaped MNs in soma within the PCL of cerebellar cortex and every unit of MNs shaped as pentagon. Every pentagon-shaped mitochondrial unit seemed to be in a 2D plane. By handling and rotating the VR, we further could find the hexagonal-shaped MNs in soma seemed to be in a 2D plane.

At the 50" of the movie, we could see 4 white line-outlined hexagonal-shaped MNs in soma within the PCL of cerebellar cortex, and every unit of MNs shaped as pentagon again. By handling and rotating the VR, we could find the hexagonal-shaped MNs in soma seemed to be in a 2D plane.

In this movie, we could number no less than 20 such hexagonal-shaped MNs, which although located in the different directions, surrounded around the empty core which should be nuclei of PCs.

Total duration of the movie was 1'13".

Movie 4. Primary immersive VR of MNs in axons of PCs.

All 141 images were corresponding to Figs. 3G-3I, in which the 4 axons were marked as a1 - 4.

From the 1" to 22" of the movie, the dashed line outlined axon 3. The appeared handle in the picture indicated the liner axons originating from the bottom of soma and sending through to the GCL layer. The total length was about 15 μm.

From the 23" to 33" of the movie, the dashed line outlined axon 4. The appeared handle in the picture indicated the liner axons branched from the shaft of axon 3 and sending through to the GCL layer. The total length was about 15 μm.

From the 34" to 46" of the movie, the picture was dragged even closer to enlarge axons 3 and 4.

From the 47" to 1'05" of the movie, we could seen the axon 3 contained bright green mitochondria-like slender structures (i.e. mitochondrial mesh described in the following parts), in addition, these structures connected up and down, which made the MNs in axons of PCs looking like a string of knots one by one.

From the 1'06" to 1'25" of the movie, we could seen the axon 1 outlined by dashed white line. The appeared handle in the picture retrogradely indicated the liner axons sending through to the GCL layer and originated from the bottom of PC's soma. The bright green slender MNs connected up and down and formed a string of green knots. The total length was about 30 μm.

From the 1'26" to end of the movie, we could seen theshorter axon 2 outlined by dashed white line. The bright green slender MNs connected up and down and formed a string of green knots. The total length was only about 10 μm.

Total duration of the movie was 1'46".

Movie 5. Secondary immersive VR with Aivia's AI-powered segmentation but without classifiers of meshed MNs in dendritic trees of PCs.

All 89 images were corresponding to Figs. 3A-3C.

The left side of the movie showed the primary VR; the right side showed the secondary VR after Aivia's AI-powered segmentation but without classifiers, which were corresponding to Fig. 4A.

It could be achieved to drag, enlarge and even step into the meshed MNs. We could see different colors of did not indicate the size of mitochondrial meshes within the dendritic trees of PCs, but represented the segmentation of meshes. We observed the meshes connected head to end along the long axis, so formed long chains.

From the 2'35" to 2'38" of the movie, the dashed line outlined and the handle pointed to one section of chain-like mitochondrial mesh with the length of about 10 μm.

From the 2'42" to 3'15" of the movie, the dashed line outlined a longer section of chain-like mitochondrial mesh with the length of about 50 μm.

From the 3'43" to 3'58" of the movie, three dashed circles outlined three tiny branches of mitochondrial mesh which appeared as small spines-like structures and we guessed they might be dendrite spines. There were so many such kind of spine-like mitochondrial mesh branches in the movie at 4'5", 4'10", 4'20", 4'28", 4'32", 4'47", 5'41", 5'56", 6'04", .

From the 5'07" to 5'15" of the movie, the dashed line outlined a short section of chain-like mitochondrial mesh with the length of about 7 μm.

From the 6'21" to the end of the movie, the VR observer stepped into the somatic body and found the mitochondrial meshes connected in a large area and formed the sponge-like structures. It was empty in the heart of the soma which should be the location of nuclei.

Total duration of the movie was 7'50".

Movie 6. Secondary immersive VR with both Aivia's AI-powered segmentation and classifiers of meshed MNs in dendritic trees of PCs.

All 89 images were corresponding to Figs. 3A-3C. The left side of the movie showed the primary VR; the right side showed the secondary VR after Aivia's AI-powered segmentation and classifiers, which were corresponding to Fig. 4B and Movie 5. All mitochondrial meshes in the dendritic trees of PCs were given a dark green pseudo-color, and the mitochondrial meshes in the soma of PCs were given a lake blue pseudo-color. We further calculated the parameters of surface area and volume of every mitochondrial mesh by using this data.

Total duration of the movie was 1'46".

Movie 7. Secondary immersive VR with Aivia's AI-powered segmentation but without classifiers of meshed MNs in soma of PCs.

All 93 images were corresponding to Figs. 3D-3F.

It showed the secondary VR after Aivia's AI-powered segmentation but without classifiers, which were corresponding to Fig. 4C.

We observed the mitochondrial meshes within the somatic bodies connected into pieces and formed a sponge-like structure. It was interesting to find that these meshes looked like a jigsaw puzzle due to their different colors and shapes. Each mitochondrial mesh could be seen as a fundamental unit which owned a main body and sent to no less than 5 short branches to different directions for further connecting.

Total duration of the movie was 3'26".

Movie 8. Secondary immersive VR with both Aivia's AI-powered segmentation and classifiers of meshed MNs in soma of PCs.

All 93 images were corresponding to Figs. 3D-3F. The left side of the movie showed the primary VR; the right side showed the secondary VR after Aivia's AI-powered segmentation and classifiers, which were corresponding to Fig. 4D and Movie 7. All mitochondrial meshes in the dendritic trees of PCs were given a purple pseudo-color, and the mitochondrial meshes in the soma of PCs were given a lake blue or deep red dark greenpseudo-color. We further calculated the parameters of surface area and volume of every mitochondrial mesh by using this data.

Total duration of the movie was 1'42".

Movie 9. Secondary immersive VR with Aivia's AI-powered segmentation but without classifiers of meshed MNs in axons of PCs.

All 141 images were corresponding to Figs. 3G-3I.

It showed the secondary VR after Aivia's AI-powered segmentation but without classifiers, which were corresponding to Fig. 4E.

It showed the mitochondrial meshes in the axons could be divided into two types: one type was named as silk-like axons because the mitochondrial meshes connected to each other just like them in dendritic trees; another type was named as granular-like axons because they were distributed as an isolated island.

Total duration of the movie was 3'09".

Movie 10. Secondary immersive VR with both Aivia's AI-powered segmentation and classifiers of meshed MNs in axons of PCs.

All 141 images were corresponding to Figs. 3G-3I. The left side of the movie showed the primary VR; the right side showed the secondary VR after Aivia's AI-powered segmentation and classifiers, which were corresponding to Fig. 4F and Movie 9. All mitochondrial meshes in the soma of PCs were given a deep red pseudo-color, and the silk-like mitochondrial meshes in the axons were given a dark green pseudo-color, and the granular-like mitochondrial meshes in the axons were given a yellow pseudo-color. We further calculated the parameters of surface area and volume of every mitochondrial mesh by using this data.

Total duration of the movie was 2'41".
